
JOURNAL INFORMATION
==============================
NLM Title Abbreviation: ScientificWorldJournal
Journal ID: ScientificWorldJournal
NLM Journal ID: 101131163
Journal ID: TSWJ

The Scientific World Journal

ISSN: 2356-6140
EISSN: 1537-744X
Publisher: Wiley

ARTICLE INFORMATION
==============================
PMCID: PMC6009239
PMID: 29973831
DOI: 10.1100/tsw.2002.56
Article ID: 175353
Article version: 1
Subjects: Short Report

Crystal Structure of Murine Tcl1 Oncoprotein and Conserved Surface Features of the Molecules of the Tcl1 Family

Petock John M. 1
Torshin Ivan Y. 1
Wang Yuan-Fang 1
Du Bois Garrett C. 3
Croce Carlo M. 3
Harrison Robert W. 2
Weber Robert T. 1 *
Departments of Biology and Chemistry, Georgia State University, Atlanta, GA;, USA
Department of Computer Science, Georgia State University, Atlanta, GA, USA
Department of Microbiology and Immunology, Kimmel Cancer Center, Thomas Jefferson University, Philadelphia, PA, USA
*Robert T. Weber: iweber@gsu.edu
Publication date: 2002
Electronic publication date: 2002 Apr 19
Volume: 2
First page: 119
Last page: 120
Copyright: Copyright © 2002 John M. Petock et al.
Copyright year: 2002
License: This is an open access article distributed under the Creative Commons Attribution License, which permits unrestricted use, distribution, and reproduction in any medium, provided the original work is properly cited.
License URL: https://creativecommons.org/licenses/by/3.0/

Funding: ELSA U. PARDEE FOUNDATION National Cancer Institute

==============================
Advances in Gene Technology: The Genome and Beyond –
Structural Biology for Medicine (Proceedings of the 2002 Miami
*Nature Biotechnology* Winter Symposium)
*TheScientificWorld* 2002, 2(S2), 119–120
ISSN 1532-2246; DOI 10.1100/tsw.2002.56

### CRYSTAL STRUCTURE OF MURINE TCL1 ONCOPROTEIN AND CONSERVED SURFACE FEATURES OF THE MOLECULES OF THE TCL1 FAMILY

John M. Petock[a], Ivan Y. Torshin[a], Yuan-Fang Wang[a], Garrett C. Du Bois[c], Carlo M. Croce[c], Robert W. Harrison[b], and Irene T. Weber[*,a]
[a]Departments of Biology and Chemistry, Georgia State University, Atlanta, GA; [b]Department of Computer Science, Georgia State University, Atlanta, GA; [c]Department of Microbiology and Immunology, Kimmel Cancer Center, Thomas Jefferson University, Philadelphia, PA
[*] iweber@gsu.edu

**INTRODUCTION.** The structures of members of the Tcl1 oncoprotein family are being studied in order to understand their roles in lymphocyte biology and their development of lymphocytic diseases[1-3]. These ~15 kD proteins share 25–80% sequence identity between the members of the family. No sequence similarity was found with other human genes suggesting a unique cellular role(s). Family members share an uncommon tertiary structure of an eight-stranded beta barrel. Recently, the crystal structure of murine Tcl1 was determined[3]. The three structures were analyzed to reveal conserved features indicative of a potential binding site for an interacting protein.

**METHODS.** Murine Tcl1 crystals were prepared as described[3]. X-ray diffraction data were collected on a Quantum 4 detector at beamline X12B of the National Synchrotron Light Source at Brookhaven National Laboratories. The structure was solved using a homology model based on the human Tcl1 structure. The crystals showed pseudo-merohedral twinning that generated a pseudo-*I*222 symmetry. Therefore, the structure was refined with SHELX97 to handle crystal twinning[4].

**RESULTS.** The mTcl1 structure consists of a dimer in the C2 space group with $R = 0.226$, $R$-free $= 0.236$. The crystal structures of mTcl1, hTcl1, and hMtcp1 are very similar (Fig. 1). Human and murine Tcl1 have an RMSD of ~0.6 Å for 100 Cα atoms, while murine Tcl1 and human Mtcp1 show ~0.5 Å RMSD for 97 Cα atoms. The three protein structures have high internal symmetry from a tandem repeat with RMSD ranging from 1.1–1.7 Å despite low sequence identity of 12–13%. All three structures share a conserved planar surface that may be involved in protein-protein interactions.

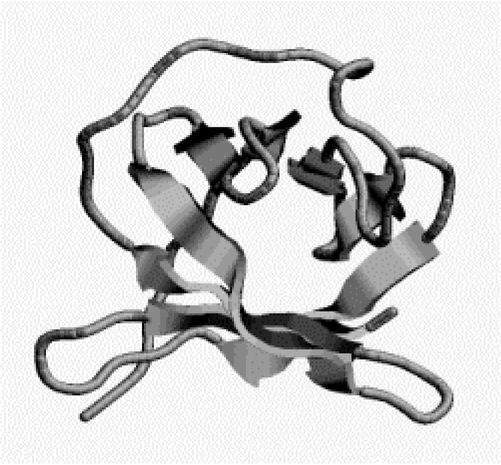

**FIGURE 1**.

**DISCUSSION**. Tcl1, Tcl1b, and Mtcp1 were shown to interact with the protein kinase Akt, which plays a key role in proliferation and survival of lymphocytes[5]. Comparison of the structures has defined common regions as potential binding sites for interacting proteins that modulate the cellular function of these unique oncoproteins. The Tcl1 family share very similar tertiary structures with a common planar surface. However, the planar surfaces of Tcl1 and Mtcp1 differ in the charge distribution, suggesting that they do not bind the same protein or bind it in different orientations.

**ACKNOWLEDGMENTS.** Many thanks to Dr. B. Mahalingam, Dr. G. Sheldrick, and Dr. R. Herbst-Irmer. This work was supported by the Elsa U. Pardee Foundation and the National Cancer Institute grant CA76259.

**REFERENCES**

1. Fu, Z.Q., Du Bois, G.C., Song, S.P., Kulikovskaya, I., Virigilio, L., Croce, C.M., Weber, I.T., and Harrison, R.W. (1998) Proc. Natl. Acad. Sci. U. S. A. 95, 3413–3418.
2. Hoh, F., Yang, Y.S., Guignard, L., Padilla, A., Stern, M.H., Lhoste, J.M., and van Tilbeurgh, H. (1998) Structure 6, 147–155.
3. Petock, J.M., Torshin, I.Y., Wang, Y.F., Dubois, G.C., Croce, C.M., Harrison, R.W., and Weber, I.T. (2001) Acta Crystallogr. D57, 1545–1551.
4. Herbst-Irmer, R. and Sheldrick, G.M. (1998) Acta Crystallogr. B54, 443–449.
5. Pekarsky, Y., Koval, A., Hallas, C., Bichi, R., Tresini, M., Malstrom, S., Russo, G., Tsichlis, P., and Croce, C.M. (2000) Proc. Natl. Acad. Sci. U. S. A. 97, 3028–3033.
